# Atypical Periprosthetic Joint Infection Post-Total Knee Arthroplasty: A Case of Actinomyces europaeus

**DOI:** 10.7759/cureus.70973

**Published:** 2024-10-07

**Authors:** Razvan Spiridonica, Mihnea Popa, Adrian Cursaru, Georgian L Iacobescu, Mihai Nica, Mihai A Costache, Sergiu Iordache, Bogdan Serban, Bogdan Cretu

**Affiliations:** 1 Orthopedics and Traumatology Department, University Emergency Hospital Bucharest, Bucharest, ROU; 2 Orthopedics and Traumatology Department, Carol Davila University of Medicine and Pharmacy, Bucharest, ROU; 3 Orthopedics and Traumatology Department, University Emergency Hospital Bucharest, Bucuresti, ROU

**Keywords:** actinomyces europaeus, maldi-tof ms, periprosthetic joint infection, rare pathogens, total knee arthoplasty

## Abstract

Periprosthetic joint infection (PJI) is a significant complication following total knee arthroplasty (TKA), representing a substantial challenge due to the difficulty in diagnosis and management. The main causes are predominantly common bacteria, but rare pathogens such as *Actinomyces europaeus* can complicate diagnosis and treatment. We report a unique case of a 75-year-old Caucasian patient with a history of multiple comorbidities including obesity, arterial hypertension, total thyroidectomy, rheumatoid arthritis, and prior venous thrombosis. The patient presented with pain, functional impairment, and signs of inflammation in the left knee seven months post-TKA. An active fistula was also noted. Initial management with broad-spectrum antibiotics did not halt the progression of infection, prompting further diagnostic evaluation. *Actinomyces europaeus* was identified as the causative agent through cultures and matrix-assisted laser desorption/ionization time-of-flight mass spectrometry (MALDI-TOF MS). A two-stage surgical intervention was necessitated, involving removal of the infected prosthesis followed by reimplantation with an antibiotic-impregnated spacer. This case highlights the importance of considering rare organisms like *Actinomyces europaeus* in atypical PJI. It underscores the necessity of advanced diagnostic tools and susceptibility testing in managing infections effectively. Timely intervention, tailored antimicrobial therapy, and appropriate surgical strategies are crucial for successful outcomes in infections involving uncommon pathogens.

## Introduction

Periprosthetic joint infection (PJI) is a serious complication following arthroplasty that imposes substantial burdens on healthcare systems in terms of resources and costs. In recent years, significant advancements have been made in the diagnosis and treatment of PJI, particularly in identifying the specific pathogens involved to facilitate targeted antibiotic therapy. According to available data, the occurrence of periprosthetic joint infection in total knee arthroplasties (TKA) is estimated to be approximately 0.8% to 1.9% [1-3]. It is worth noting that cases of joint infection caused by *Actinomyces europaeus* are rare.

The most frequently reported anaerobes causing periprosthetic joint infection are *Cutibacterium acnes* and *Bacteroides *spp. [4]. PJIs attributed to *Actinomyces *spp. are rarely documented in the literature, with limited information available on their clinical features, treatment, and outcomes. Actinomyces is a gram-positive, facultatively anaerobic bacterium commonly found as a commensal in the human oral cavity. It is also known to colonize the skin and the gastrointestinal, respiratory, and genitourinary tracts [5]. A joint infection with Actinomyces, specifically *Actinomyces europaeus*, is a rare but serious condition. Joint infections can occur as a result of direct spread from nearby structures or through hematogenous dissemination. Infections of the bone and joints due to Actinomyces spp., such as osteomyelitis of the jaw, tibia, foot, pubic symphysis, and vertebral osteomyelitis, have been reported [5,6].

According to Dagher et al. [7], the main symptoms of Actinomyces infection associated with prosthetic joint infections include chronic joint pain, which was reported in 91% of the cases, drainage or sinus tract formation observed in 63% of the patients, erythema indicating inflammation in some cases, and swelling commonly noted around the infected joint area. In some cases, purulent aspirate was found during joint aspiration. Diagnosis is typically made through a combination of clinical evaluation, imaging studies (such as X-rays or MRI), and laboratory tests, including joint fluid analysis. While *Actinomyces europaeus* is a bacterium that can cause infections, it is important to note that the presence of *Actinomyces europaeus* alone does not necessarily increase the risk of infection. Infections with Actinomyces species typically occur when there is a breach in the body's natural barriers, such as the skin or mucous membranes, allowing the bacteria to enter and cause an infection [8, 9].

Factors that may increase the risk of *Actinomyces europaeus* infection include 1) weakened immune system: individuals with compromised immune systems, such as those with HIV/AIDS, undergoing chemotherapy, or taking immunosuppressive medications, may be more susceptible to Actinomyces infections; 2) dental procedures: dental procedures that involve manipulation of the oral cavity, such as tooth extractions or root canals, can create an opportunity for *Actinomyces europaeus* to enter the bloodstream and potentially cause an infection; 3) trauma or surgery: any trauma or surgical procedure that disrupts the skin or mucous membranes can provide an entry point for *Actinomyces europaeus* and increase the risk of infection; 4) underlying medical conditions: certain medical conditions, such as diabetes or chronic lung disease, may weaken the body's defenses and make individuals more susceptible to Actinomyces infections [3, 6, 9].

Several studies have indicated that obesity can increase the likelihood of infection, although not all studies have reached the same conclusion. The commonly used threshold for measuring obesity is a body mass index (BMI) of 35. It is possible that longer surgical procedures and the presence of other health conditions contribute to the increased risk of obesity. However, even after accounting for other factors in various studies, obesity has consistently been identified as an independent risk factor [10]. Conversely, another study suggested that a low BMI of 25, which may indicate nutritional deficiencies, immunosuppression, or underlying rheumatoid arthritis, was associated with an increased risk of PJI [11]. In cases of rheumatoid arthritis, joint replacement surgery is often performed, and these patients have been found to have a higher baseline risk of infection compared to the general population. Additionally, the use of immunosuppressive medications to treat rheumatoid arthritis may further increase the risk of infection. To improve the assessment and development of preoperative prevention strategies, it would be beneficial to identify patients with rheumatoid arthritis who are at a higher risk of prosthetic joint infections compared to those without the condition. In fact, within the first year, the infection rate in rheumatoid arthritis patients has been reported to be as high as 2.3% [12].

We present a case of prosthetic joint infection (PJI) caused by a rare species of Actinomyces. To the best of our knowledge, this report represents the first case description with *Actinomyces europaeus* as the causative microorganism for PJI. This case underscores the importance of thorough analysis and interpretation of clinical findings, which may reveal a specific and often overlooked source of contamination.

## Case presentation

**Figure 1:**
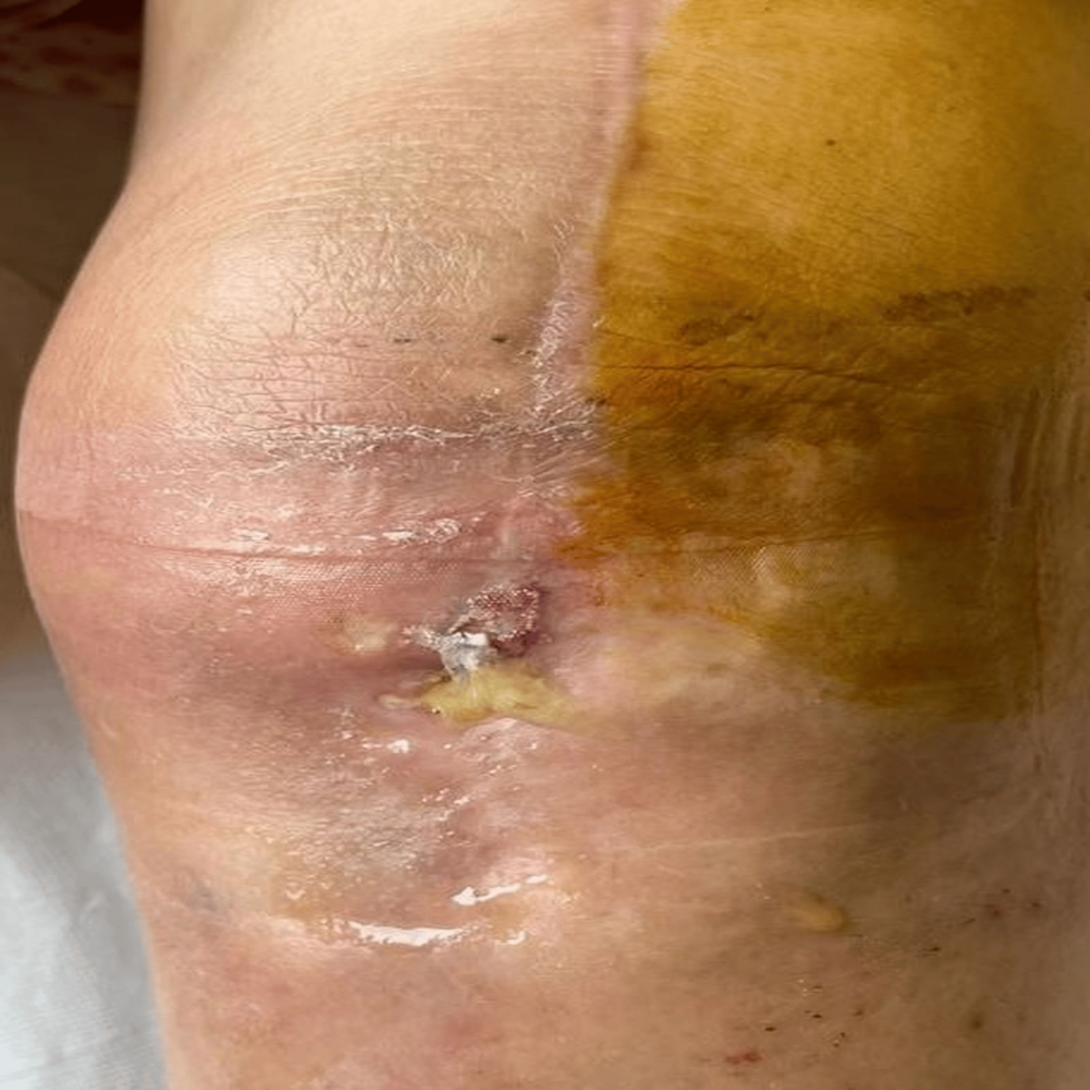
Fistula following total knee arthroplasty Postoperative knee of a patient who had total knee arthroplasty (TKA) and later developed a septic evolution. This photograph, captured two months after the beginning of ongoing knee pain, displays a fistula on the front of the knee, accompanied by redness, swelling, and purulent fluid, suggesting the presence of an infection.

We present the case of a 75-year-old female patient who presented to the emergency unit in the Orthopedics and Traumatology service for pain in the left knee, functional impotence, with inflammatory signs present and active fistula at the same level, seven months after left total knee arthroplasty and after two months of septic evolution (Figure 1).

Based on the patient's medical history, the individual underwent a total knee arthroplasty on the right side in 2016 and has been diagnosed with grade I obesity and arterial hypertension. The patient has also undergone a total thyroidectomy and is currently being treated with Euthyrox. Additionally, rheumatoid arthritis was diagnosed in the same year, 2016, and is being managed with methotrexate. The patient's medical history further includes a deep vein thrombosis in the left lower limb, also occurring in 2016, and a past event of intestinal occlusion in 1995. This comprehensive medical background provides essential context for understanding the patient's current health status and medical needs.

At admission to our department, initial blood tests showed anemia (8.9 g/dL), increased levels of erythrocyte sedimentation rate (ESR) (50 mm/h), C-reactive protein (4.4 mg/dL), fibrinogen (624 mg/dL), low iron levels (37 ug/dL) and normal glucose values. Samples are collected from the knee fistula, joint fluid, Gram stain, Ziehl-Neelsen stain, pharyngeal and nasal exudate, subsequently with negative results. Double antibiotic therapy with vancomycin and second-generation cephalosporin (cefuroxime) was started for preoperative preparation. An interdisciplinary cardiology consultation is performed that does not identify an acute cardiac pathology that could contraindicate surgical intervention (BP=160/80 mmHg under antihypertensive treatment with amlodipine, metoprolol, propafenone, ramipril). In most instances, antibiotics alone do not suffice to treat periprosthetic joint infections.

Typically, these infections require surgical approaches including irrigation and debridement, one-stage reimplantation, two-stage reimplantation, resection arthroplasty, or amputation to effectively manage the condition [13]; in our case, the surgical team decided on a two-stage reimplantation based on the patient's condition. However, two-stage reimplantation is commonly considered the optimal strategy for addressing periprosthetic joint infections [14]. On a similar note, one-stage revision, which encompasses irrigation, debridement, and reimplantation in a single surgical session, is recommended only under certain conditions. These conditions include the identification of the causative organism with susceptibility to gram-positive antibiotics, the feasibility of administering antibiotic treatment for 12 weeks, an infection that is not polymicrobial, and the patient's overall readiness for surgery, characterized by adequate soft tissue coverage, sufficient bone stock for reconstruction, and no significant immunosuppression or other serious health issues [13].

**Figure 2:**
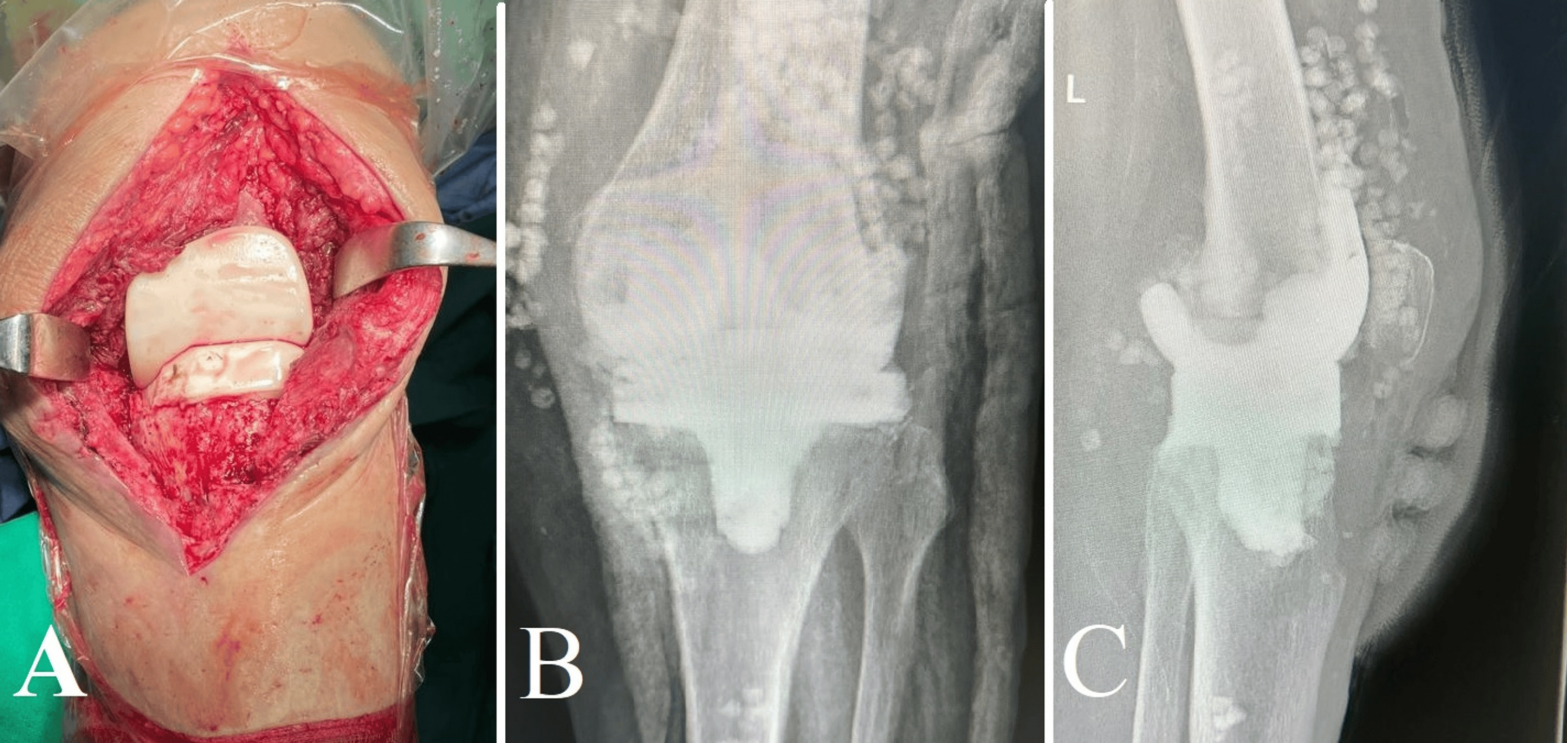
Surgical intervention and post-operative imaging following two-stage exchange arthroplasty for septic total knee arthroplasty (A) Surgical perspective displaying the extraction of the contaminated prosthesis and the insertion of a spacer infused with antibiotics. (B) Postoperative anteroposterior (AP) X-ray displaying the location of the spacer and calcium sulfate beads infused with antibiotics. (C) Postoperative lateral X-ray showing the spacer and beads in their anatomical position.

After completing preoperative preparations on April 6, 2023, the surgical team carried out the removal of the infected knee prosthesis, performed a two-stage exchange arthroplasty including the complete removal of the knee prosthesis, and conducted thorough debridement of the devitalized tissues along with an extensive chemical clean-up. Subsequently, an antibiotic-impregnated spacer and antibiotic-impregnated calcium sulfate beads were implanted (Figure 2). These act as carriers for the antibiotics, allowing for their gradual dissolution and release. This targeted delivery method ensures high concentrations of antibiotics directly at the infection site, thereby enhancing the efficacy of the treatment while reducing the risk of systemic exposure and associated side effects.

**Figure 3:**
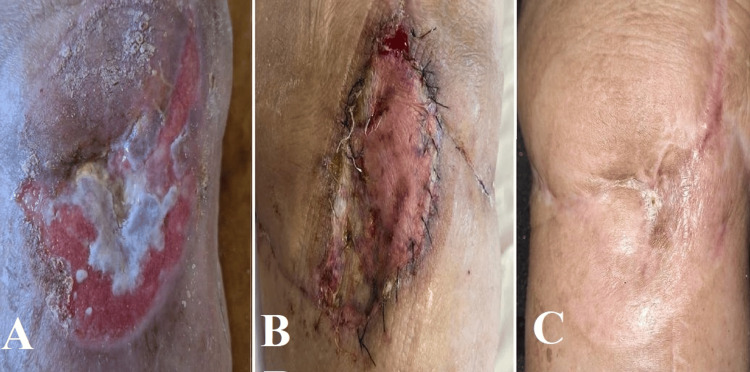
Sequential evolution of skin condition and healing following orthopedic and plastic surgery interventions after septic total knee arthroplasty. (A) Initial poor healing with erythema and necrosis, indicating persistent infection. (B) Postoperative image following a combination of orthopedic and plastic surgery involving the use of a skin flap to enhance blood flow and promote healing. (C) Within eight months after the surgery, the knee shows full epithelialization and little scarring, which suggests effective control of infection and treatment of the incision.

Samples were collected intraoperatively and later analyzed, revealing an infection with *Actinomyces europaeus*, which showed sensitivity to benzylpenicillin, piperacillin/tazobactam, meropenem, vancomycin, and chloramphenicol. Due to an unfavorable local postoperative response, a negative pressure wound therapy system was installed. On April 21, 2023, a further surgical procedure was conducted by a multidisciplinary team, including a plastic surgery component that involved autologous skin grafting (Figure 3).

The treatment regimen was gradually scaled down, with the patient undergoing continuous clinical and biological monitoring. This approach led to a favorable outcome locally, marked by a reduction in the levels of inflammatory markers Table 1.

**Table 1:** Dynamic monitoring of biological parameters This table shows the patient's key biological parameters measured at different stages during treatment, starting from the initial admission on March 22, 2023, through to April 30, 2023. Notable trends include the reduction in C-reactive protein (CRP) and erythrocyte sedimentation rate (ESR), indicating a decrease in inflammation, along with an improvement in hemoglobin (HBG) and iron levels, reflecting the patient's recovery progress.

	03.22.2023	03.28.2023	04.20.2023	04.30.2023
CRP	4.4 mg/dL	6.39 mg/dL	3,84 mg/dL	1.34 mg/dL
ESR	50 mm/h	50 mm/h	-	10 mm/h
Fibrinogen	624 mg/dL	409 mg/dL	420 mg/dL	280 mg/dL
HBG	8.9 g/dL	8.4 g/dL	12,8 g/dL	12,1 g/dL
Iron	37 ug/dL	14.4 ug/dL	-	56 ug/dL

The pathological specimens collected consisted of intraoperative fluid, tissue fragments, and biopsies containing purulent secretion. While microscopic analysis did not objectively show the presence of microorganisms, cultures revealed a single microorganism present in moderate quantities. This organism was identified as *Actinomyces europaeus* through the use of the matrix-assisted laser desorption/ionization time-of-flight mass spectrometry (MALDI-TOF MS; Bruker Microflex LT model, Bruker Daltonics, Billerica, Massachusetts) technique. MALDI-TOF MS is a method for identifying biomolecules according to their mass-to-charge ratio. A sample is combined with a matrix material that facilitates ionization upon exposure to a laser. The resultant ions are accelerated, and their period of flight is monitored, facilitating the determination of the molecules' mass. This method is extensively employed for swift microbiological identification and protein analysis owing to its precision and efficacy.

The cell wall structure of the microorganisms to be identified determines how to prepare the samples for this system. The advantages of MALDI-TOF MS over conventional microbiology methods include its speed, accuracy, and cost-effectiveness. It can yield results within minutes, compared to hours or days, with traditional methods, such as biochemical testing or molecular methods. This leads to a faster turnaround time for clinicians, enabling them to make prompt and appropriate therapeutic decisions, particularly in the management of infectious diseases where time is of the essence. Despite its benefits, certain limitations of the MALDI-TOF MS technology need to be considered. Its performance heavily relies on the quality and completeness of the reference database. In addition, it might struggle to differentiate between closely related species or strains. Furthermore, it cannot provide antibiotic susceptibility data, which is crucial in guiding antimicrobial therapy.

MALDI-TOF MS, a cutting-edge microbiological diagnostics instrument, was used to identify the Actinomyces infection in this instance. After 48 hours of incubation on Columbia blood agar, a pinpoint colony was transferred to a MALDI-TOF MS target plate and covered with a matrix, Cyano-4-hydroxycinnamic acid. After allowing the matrix to dry, the sample was analyzed using Bruker autoFlex Speed and BioTyper MBT Compass version 4.1. The identification score of 2.18 confirmed *Actinomyces europaeus* as the pathogen.

Determining the antimicrobial susceptibility profile was the crucial next step in managing this infection. This was accomplished by testing Brucella Blood Agar for antibiotic susceptibility under anaerobic conditions using E-test bands (manufactured by bioMérieux). By measuring the Minimum Inhibitory Concentration (MIC) of antibiotics, the E-test allows for the quantitative determination of antibiotic susceptibility. The MIC results, expressed in mg/L, were interpreted based on Clinical and Laboratory Standards Institute (CLSI) recommendations from 2023. These recommendations provide a standardized method for interpreting and applying antimicrobial susceptibility test results, thereby facilitating the selection of the most suitable antimicrobial therapy. This methodical approach to diagnosis and susceptibility testing allowed for the targeted treatment of this Actinomyces infection.

On blood agar, colonies of *Actinomyces europaeus* appear as microscopic, translucent, or colorless points. These colonies may appear granular or powdery but are typically smooth and spherical. Around the colonies, they may develop a yellow or ochre pigmentation [3,5]. On chocolate agar, *Actinomyces europaeus* colonies may have a yellow or ochre hue, which is frequently more noticeable and pronounced than on blood agar. These colonies have a filamentous structure and the ability to form distinct radial sides. The chromogenic medium can be utilized to identify *Actinomyces *species based on their ability to metabolize specific substances. *Actinomyces europaeus* typically demonstrates negligible chromogenic activity on standard chromogenic media. It is crucial to note that the morphological descriptions of bacterial cultures can vary depending on the culture conditions and media employed and that the appearance of the colonies can also be affected by other variables, such as incubation time and growth temperature. However, it can become pathogenic and cause infections, especially in situations involving trauma or surgical procedures [3,9]. 

*Actinomyces europaeus* is susceptible to the antibiotics benzylpenicillin, piperacillin/tazobactam, meropenem, vancomycin, and chloramphenicol, according to the results. However, it demonstrates intermediate clindamycin and metronidazole resistance. This information indicates that *Actinomyces europaeus* is the cause of the patient's joint infection, and the treatment should be selected based on the bacteria's susceptibility to antibiotics. Depending on the sensitivity profile of the bacteria and the susceptibility test results, antibiotics such as benzylpenicillin, piperacillin/tazobactam, meropenem, or vancomycin may be utilized as therapeutic options. It is essential to observe that antibiotic susceptibility data are interpreted in accordance with CLSI 2023 standards, which provide guidelines for interpreting minimum inhibitory concentration (MIC) values [5, 13].

## Discussion

Total joint arthroplasty (TJA) can result in significant complications, such as early and delayed infections, primarily due to perioperative bacterial colonization. However, most late infections result from hematogenous dissemination from endogenous sources. Diabetes mellitus, post-traumatic osteoarthritis, prior surgery, chronic liver disease, corticosteroid therapy, and intravenous substance abuse are identified as risk factors. *Actinomyces europaeus*, a prevalent anaerobic bacterium with low pathogenicity that inhabits the human body, has infrequently been associated with TJA infections [7, 15, 16].

To reduce infection risks in total joint arthroplasty, proactive measures across various surgical phases are crucial. Pre-operatively, managing reversible comorbidities like diabetes, obesity, and malnutrition is vital, which includes optimizing blood glucose levels and nutritional status. During the procedure, strict aseptic techniques are essential, involving prophylactic antibiotics before the incision and applying antiseptic solutions such as chlorhexidine. Efforts should focus on minimizing operating time and reducing room traffic to lower contamination risks. Post-operatively, vigilant wound care, prompt infection signs treatment, and rigorous hygiene are fundamental [10].

Identifying *Actinomyces *or related species has proven challenging with biochemical tests [5]. However, differences in antimicrobial susceptibility profiles among species suggest that accurate identification can influence clinical outcomes, making it a critical yet challenging task. Significant advances in molecular diagnostic techniques have significantly improved the detection of *Actinomyces *spp. The sequencing of ribosomal RNA (rRNA) genes offers high sensitivity and specificity, allowing for accurate species identification. In addition, MALDI-TOF has emerged as a valuable instrument for rapid and accurate identification, significantly contributing to enhanced patient outcomes due to the chronic and apathetic nature of *Actinomyces *spp.-caused PJIs, a comprehensive approach is required for their treatment. Despite being primarily susceptible to beta-lactam antibiotics, their inherent ability to form biofilms and coexist with other pathogens can complicate the management of infections. The use of MALDI-TOF/MS has enabled the successful identification of most species quickly, cost-effectively, and more straightforwardly compared to traditional phenotypic methods [17-19]. Alternatively, sequencing the 16S rRNA gene offers a method with higher precision [20].

MALDI-TOF MS has emerged as a crucial instrument in clinical microbiology, facilitating the swift and precise identification of harmful microorganisms. The method provides substantial benefits in infection diagnosis by contrasting protein profiles of organisms with a comprehensive library of established microbial spectra. MALDI-TOF MS was crucial in finding *Actinomyces europaeus*, a slow-growing organism that could have been overlooked or incorrectly identified. Nonetheless, like any technology, there are constraints. The accuracy of the identification is largely dependent on the quality of the database. In the absence of the microorganism in the database, the system may yield no results or propose a similarly related organism, which could result in a false-positive diagnosis. This underscores the necessity for a proficient specialist to analyze the results and prevent misdiagnosis, especially in intricate circumstances such as this. Notwithstanding its limits, the advantages of MALDI-TOF MS, including rapidity, cost-efficiency, and precision in identifying a diverse array of organisms, render it an essential instrument in infection management. It enabled the prompt diagnosis and suitable treatment of the patient, highlighting its clinical significance [21]. 

Two-stage exchange arthroplasty, which consists of resection arthroplasty and staged reconstruction with a cemented spacer, has proven effective in controlling infections and minimizing complications. Antibiotic-impregnated cement spacers facilitate the local delivery of antimicrobials to soft tissues surrounding the joint, achieving concentrations that are difficult to reach with systemic antibiotics. Although there are recommendations to use specific antibiotics in the spacer for the specific pathogen identified [22], vancomycin and aminoglycosides are still the most frequently utilized. These broad-spectrum antibiotics are preferred due to their thermal stability and negligible impact on the mechanical integrity of the cement [23, 24]. In our case, due to the uniform susceptibility of *Actinomyces* spp. to vancomycin, a vancomycin-impregnated spacer is frequently recommended. Based on known active or in vitro susceptibilities, a suggested regimen comprises a minimum of six weeks of highly bioavailable oral or parenteral beta-lactam antibiotics. For beta-lactam-intolerant patients, tetracycline (if susceptible) and vancomycin are viable alternatives. The function of long-term antimicrobial suppression following a two-stage exchange is a subject of ongoing debate and must be evaluated on a case-by-case basis. Generally, a course of oral amoxicillin or penicillin VK lasting between six weeks and three months is advised following reimplantation [13].

Similarly to our case, in a case report by Pina et al. [25], three synchronous periprosthetic joint infections were effectively managed through the application of a two-stage arthroplasty involving the use of high-dose antibiotic cement spacers followed by definitive reimplantation. This intervention mirrors the therapeutic approach outlined in our case, where similar management strategies were employed. Pina et al. [25] noted that while the two-stage procedure facilitated infection control, it also posed risks such as acute kidney injury (AKI) due to high antibiotic loads, a complication also observed in our case. Furthermore, their findings underscore the necessity of monitoring and adjusting treatment modalities in response to systemic side effects of antibiotic therapy, reflecting the challenges we encountered in balancing infection eradication with minimizing adverse effects. This parallel underlines the critical need for vigilant post-operative monitoring and potential adjustments in antibiotic management to mitigate the risks of systemic toxicity, especially in cases involving high-risk patients with multiple joint replacements [25].

*Actinomyces europaeus*, not previously identified, is now known to cause necrotizing fasciitis and other musculoskeletal system infections. Research has documented *Actinomyces europaeus* strains with resistance to antibiotics such as piperacillin-tazobactam, linezolid, clindamycin, ciprofloxacin, and ceftriaxone [8]. However, all strains remained susceptible to carbapenems, vancomycin, aminopenicillins, doxycycline, tigecycline, and penicillin G. Despite this susceptibility, the increasing resistance profile of *Actinomyces europaeus* is concerning, particularly for the treatment of necrotizing soft tissue infections (NSTI). *Actinomyces europaeus* is characterized by slow growth, often taking five days or more to develop [9].

The challenges of *Actinomyces *infections extend beyond drug resistance. Many hospitals lack the capability for anaerobic antibiotic susceptibility testing, despite the cultivation of *Actinomyces*. In the United States, few hospitals undertake such testing, which leads to infrequent susceptibility testing of wound culture isolates. This situation necessitates a shift towards broader-spectrum antibiotic therapies, potentially exacerbating antibiotic resistance. Therefore, it is crucial for medical institutions to implement routine, standardized anaerobic susceptibility testing. This practice would aid in identifying effective treatments and foster responsible antibiotic use. The severity of the infection and the patient's underlying conditions adversely affected her outcome. Additionally, the role of antibiotic resistance underscores the importance of thorough susceptibility testing and careful antibiotic selection in managing *Actinomyces *infections [26].

## Conclusions

This research discusses a patient who is 75 years old and has had prolonged post-arthroplasty septic evolution. The patient was diagnosed with an unusual *Actinomyces europaeus *infection. The patient presented with a number of serious co-morbidities in addition to modified blood test values that were indicative of infection. The identification of *Actinomyces europaeus* has been made easier because to developments in molecular diagnostics such as 6S rRNA gene sequencing and MALDI-TOF. These developments have occurred despite the fact that *Actinomyces europaeus* is a slow-growing organism and presents difficulties due to its resistance to common antibiotics. It was determined that a two-stage exchange arthroplasty and targeted antibiotic medication based on susceptibility testing were necessary for effective care, highlighting the need of susceptibility testing for proper treatment. This case demonstrates the changing pathogenicity of *Actinomyces europaeus*, its increasing antibiotic resistance profile, and the necessity of doing routine anaerobic susceptibility testing to achieve the best possible results for patients.
